# Towards symbiont-based visceral leishmaniosis control: *Delftia tsuruhatensis* TC1 shows anti-**Leishmania* infantum* effect in *Phlebotomus perniciosus*

**DOI:** 10.3389/fimmu.2026.1953324

**Published:** 2026-10-30

**Authors:** Marcos López-de-Felipe, Maribel Jiménez, Eva Pérez-Martínez, Ricardo Molina, Pablo Castañeda-Casado, Joel Lelièvre, Inés Martín-Martín, Janneth Rodrigues

**Affiliations:** 1 Laboratory of Medical Entomology, National Centre for Microbiology, Instituto de Salud Carlos III, Madrid, Spain; 2 Department of Microbiology and Parasitology, Faculty of Pharmacy, Universidad Complutense de Madrid, Madrid, Spain; 3 Centro de Investigación Biomédica en Red de Enfermedades Infecciosas (CIBERINFEC), Instituto de Salud Carlos III, Madrid, Spain; 4 Global Health Medicines R&D, GSK, Madrid, Spain

**Keywords:** biological control, harmane, microbiota, *Pantoea dispersa*, vector control

## Abstract

**Introduction:**

Visceral leishmaniosis caused by **Leishmania* infantum* is a grave disease solely transmitted by vector sand flies with a high risk of mortality when left untreated. The introduction of symbiont bacteria with anti-parasitic properties within vector species represents a promising biological control strategy for vector-borne diseases. In this context, the symbiont bacterium *Delftia tsuruhatensis* strain TC1 (TC1) has previously demonstrated an *in vivo* antiparasitic effect in the sand fly *Phlebotomus duboscqi* against **Leishmania* major*, the causative agent of cutaneous leishmaniosis. The current research aims to evaluate the anti-*L. infantum* effect of TC1 within *Phlebotomus perniciosus*, a major vector of visceral leishmaniosis in Southern Europe.

**Methods:**

Newly emerged *Ph. perniciosus* females were fed on a sugar meal spiked with TC1. Bacterial midgut colonization was evaluated through plating at different time points after bacteria ingestion in selective media and molecularly verified. TC1-fed sand flies were offered an *L. infantum* infectious blood meal 24 h after spiked-sugar was removed. The anti-*L. infantum* effect of TC1 was evaluated by quantifying the number of parasites per midgut and evaluating the infection rate of the stomodeal valve in comparison with control groups. Additionally, the anti-*L. infantum* effect of TC1 cell-free supernatant and harmane, a β-carboline alkaloid secreted by TC1 and previously reported to display *in vitro* anti-leishmanial activity, were evaluated *in vitro* through dose-response curves assays and by quantification of promastigotes within the midgut of sand flies. **Leishmania* infantum* viscerotropic strains were used during assays.

**Results:**

TC1 was confirmed to colonize and persist within the midgut of female *Ph. perniciosus* up to at least 21 days after ingestion. TC1-fed sand flies showed a decrease in the number of promastigotes per midgut, as well as a reduction in the infection rate of the stomodeal valve. Both harmane and TC1-supernatant showed an anti-*L. infantum* effect under *in vitro* conditions, although no reduction in parasite counts was detected within sand flies.

**Discussion/conclusion:**

TC1 reduces the proliferation and development of *L. infantum* within the vector *Ph. perniciosus*. Our results show the potential of TC1 as a promising tool for controlling *L. infantum* in visceral leishmaniosis endemic areas in the future.

## Graphical Abstract

**Figure:**
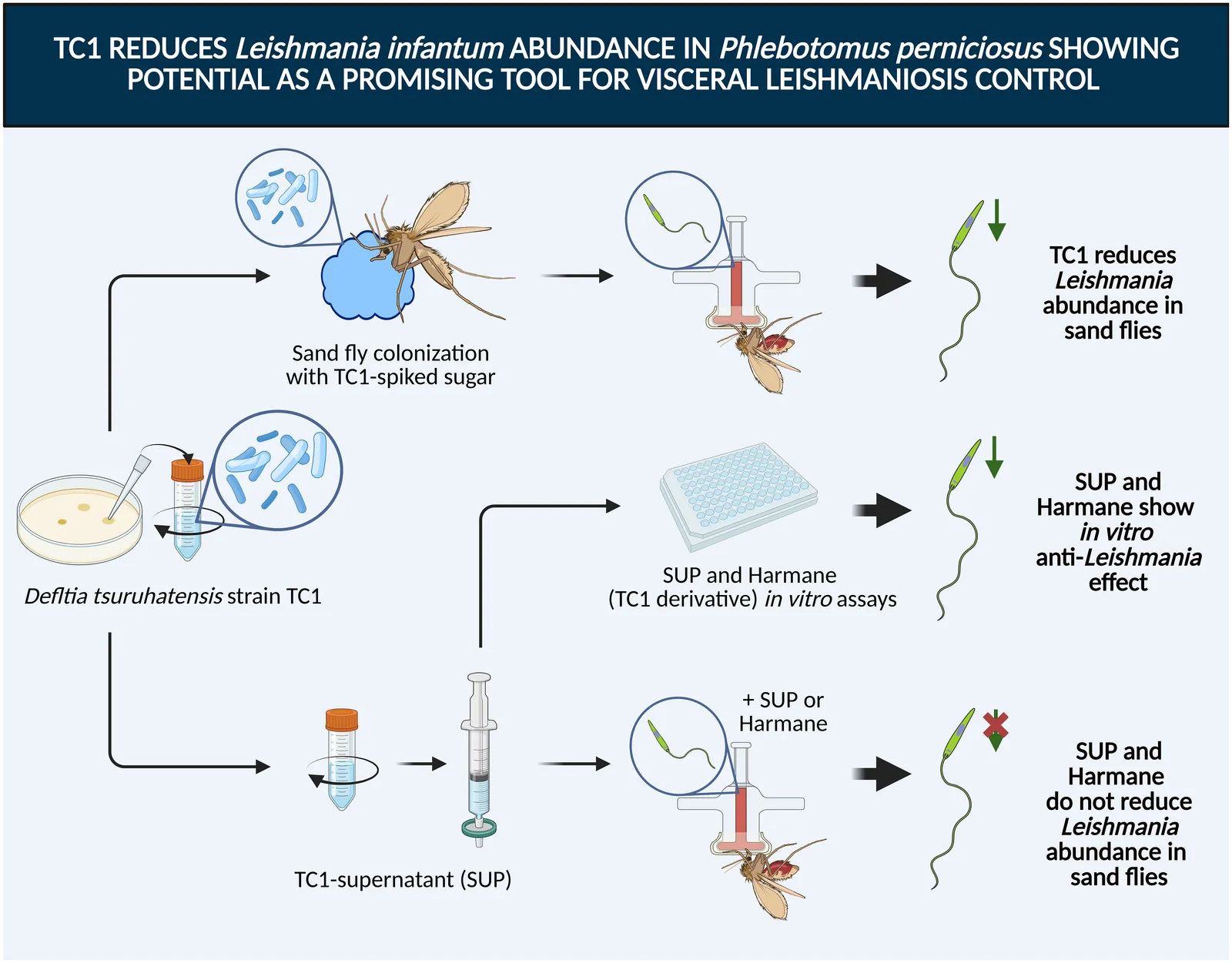

## 1 Introduction

Human leishmaniosis is a complex group of diseases caused by at least 21 species within the *Leishmania* genus (1–3). Three main clinical forms are distinguished: cutaneous leishmaniosis (CL), mucocutaneous leishmaniosis (MCL), and visceral leishmaniosis (VL), whilst other rare skin manifestations are also recognized in the literature (1, 3). Although CL is regarded as a common and generally self-limiting disease, (4), and MCL as the most disfiguring form, VL is the most severe disease caused by *Leishmania* parasites (5). Between 50.000 and 90.000 new VL cases are annually reported worldwide (6), especially in Southeast Asia and the Americas (5), while being an endemic and reemerging disease in Southern Europe (2, 7). While most infections in immunocompetent hosts are asymptomatic, severe cases are reported in infants, the elderly, and immunocompromised patients, with particular attention to *Leishmania*-HIV-coinfected patients (8). Without appropriate treatment, VL has a high fatality rate (9), being regarded as the third deadliest parasitic disease after malaria and infant cryptosporidiosis (10).

Among the different parasites causing human leishmaniosis, **Leishmania* infantum* (syn. *L. chagasi*) is the zoonotic causative agent of VL in the New World (NW) and VL and CL in the Old World (OW) (11). While dogs are the main reservoir (12), other sylvatic reservoirs such as leporids can also play a major role in certain leishmaniosis epidemiological contexts in Southern Europe (13, 14). Excluding occasional parenteral transmission through reused needles by intravenous drug users (15) or organ and blood transfusions, *L. infantum* is exclusively transmitted to humans by vector phlebotomine sand flies (3) within the *Lutzomyia* (NW) or *Phlebotomus* (OW) genera. *Phlebotomus perniciosus* is the primary vector in western Mediterranean countries such as Spain (16), where the largest VL outbreak to date in Europe was reported between 2009 and 2014 and *L. infantum* transmission still persists (17, 18).

Given the lack of a human vaccine, diagnostic challenges, treatment-related adverse effects, and the high morbidity and mortality associated with VL (3, 5, 9, 10, 19), together with the complexity of reservoir management (19), vector sand flies control is essential to tackle VL transmission (20, 21). Additionally, sand fly control faces further challenges, as breeding sites are not properly characterized, while adult resting sites are cryptic and may be highly inaccessible (16). As such, vector control programs are still mostly based on the application of aerosol insecticides (20, 21), which are highly unspecific, affecting non-target fauna and potentially leading to insecticide resistance, as has been reported previously (22). Nevertheless, while novel vector-specific control strategies such as larvicide bacteria or the sterile insect technique (SIT) are being exploited against Culicidae mosquitoes (23), similar tools for sand flies are still unavailable.

Within this context, biological control, comprising the use of selected organisms and/or their derivatives for the management of specific vectors and/or pathogens, as seen in other Diptera (24), is a promising perspective for VL control (25, 26). Specifically, midgut symbiont bacteria play a key role in *Leishmania* spp. development within vector species (27, 28), making the modification of midgut microbiota an interesting target for biological control interventions. Previous efforts within the context of CL caused by **Leishmania* major* and **Leishmania* mexicana* have reported bacteria with anti-*Leishmania* spp. effect within sand flies (i.e., *Asaia* spp. or *Ochrobactrum intermedium* and especially *Delftia tsuruhatensis* strain TC1, TC1 hereinafter), with potential for use in disease control (29–32). TC1, a Gram-negative bacteria within the ubiquitous *Delftia* genus (33), represents one of the most advanced examples of a symbiont-based intervention for vector-borne disease control. Since its original isolation from the midgut of a laboratory-reared *Anopheles stephensi* colony refractory to *Plasmodium* sp. infection, TC1 has demonstrated strong anti-*Plasmodium* effect under laboratory and semi-field conditions across multiple species of anophelines including field-collected mosquitoes, suggesting its potential for widespread use in malaria control, as supported by mathematical modeling (34).

Subsequent laboratory studies have also confirmed the anti-*L. major* effect of TC1 in *Phlebotomus duboscqi*, with complementary *in silico* modelling supporting the potential of TC1 in reducing CL transmission risk (29). In the context of VL, the potential use of transgenic bacteria for controlling *L. infantum* (26) and **Leishmania* donovani*, the causative agent of anthroponotic VL in Southeast Asia (25), has been previously proposed (25), while certain midgut bacteria have been thought to restrict the infection establishment rate of *L. infantum* in field-caught sand flies in the NW (35). However, to our knowledge, no prior studies have demonstrated a direct anti-*Leishmania* effect of symbiotic bacteria against VL-causing parasites within phlebotomine sand flies.

Here, we show that TC1 colonizes and persists in the midgut of *Ph. perniciosus* and induces a pronounced anti-*L. infantum* effect within the OW vector *Ph. perniciosus*. We further report that TC1-supernatant and harmane, a TC1 derived β-carboline alkaloid, show a dose-dependent *L. infantum* inhibition effect *in vitro*. This effect was observed against two different viscerotropic parasite strains isolated from field-caught *Ph. perniciosus*, as well as against a human clinical bone marrow isolate. These findings provide the first evidence of a bacterium inducing an anti-*L. infantum* effect within a phlebotomine sand fly vector species within the context of VL.

## 2 Methodology

### 2.1 Sand fly colonies

Two *Ph. perniciosus* sand fly laboratory colonies, established from field-collected specimens from the area of Madrid, Spain (*Ppern*-BOA: sand flies collected in Boadilla del Monte, Madrid in 1987; *Ppern*-FUE: sand flies collected in Fuenlabrada, within the context of the Madrid VL outbreak in 2012) (36, 37) were used during assays. Sand flies were kept in an environmental chamber (Binder GmbH^®^, Ref. KBW720-230V) at 27 °C, 90-100% relative humidity, and a 17 h light: 7 h dark photoperiod, and maintained as described by Molina (36). Newly emerged *Ph. perniciosus* females (1–3 days post-emergence) were used during experiments. Experimental infections were conducted using in-house-made colonization/infection containers (i.e., 9.5 x 10 cm cardboard containers covered with a mosquito net <3 mm mesh size on top and a 1.5 cm hole on the side for the introduction of mouth aspirators). Containers were placed in plastic trays with water-soaked dishcloth pieces to preserve humidity within the environmental chamber. Sand flies were kept on a regular 30% sterile sucrose diet.

### 2.2 Parasites

Three viscerotropic *L. infantum* strains were used in these studies. IPER/ES/2012/BOS1FL1 and IPER/ES/2012/POL2FL6, referred to as BOS1FL1 and POL2FL6, respectively, were isolated from naturally infected sand flies captured in the human leishmaniosis outbreak area of Madrid in 2012 as part of the vector surveillance program and were both later characterized as the MHOM/ES/87/Lombardi strain (17, 39). Additionally, MHOM/ES/2024/LLM-2544, referred to as 28840MO and characterized as *L. infantum* strain ITS-genotype A, is an anonymized clinical human bone marrow isolate obtained from the biobank collection of parasite strains of the WHO collaborating Centre for *Leishmania*sis at the National Center for Microbiology (ISCIII). **Leishmania* infantum* promastigotes were maintained on weekly passages on modified Novy-MacNeal-Nicolle (NNN) tubes with RPMI 1640 medium (Corning Inc.^®^, Ref. 10-041-CV) supplemented with 20% fetal bovine serum (Glibco^®^, Ref. 10270-106), 10 µg/ml hemin (Merck^®^, Ref. H9039), and 80 U/ml of PenStrep antibiotics (10000 U Penicillin/ml, 10000 U Streptomycin/ml; Lonza, Ref. DE17-602E) (enriched RPMI medium). NNN medium was prepared by the *Leishmania*sis and Chagas Disease Unit as a mixture of agar, NaCl, and defibrinated rabbit blood provided by Centro San Bernardo (CSB, Navarra, Spain). RPMI subcultures were prepared 2–3 days before infections to ensure parasites were in the logarithmic phase of growth at the time of the infectious blood meal.

### 2.3 Bacteria culturing and preparation

*Delftia tsuruhatensis* TC1 stocks were provided by Global Health Medicines R&D, GSK, Tres Cantos, Spain. *Pantoea dispersa*, used as a negative control, was isolated from the midgut of a *Ph. perniciosus* female from the *Ppern*-BOA laboratory reared colony, and glycerol-cryopreserved stocks were prepared and kept at -80 °C until use (unpublished data). TC1 and *Pa. dispersa* were routinely maintained in 50 µg/ml Ampicillin (Amp; Merck^®^, Ref. A9518) and Gentamicin (Gen; Merck^®^, Ref. G1272) LB-agar plates and non-selective LB-agar plates, respectively, at 28 °C. Isolated colonies were picked with a pipette tip and resuspended in 5 ml of LB broth within a 50 ml conical Falcon tube for culturing on a rotatory shaker at 200 rpm and 28 °C for 16 h. Overnight cultures were centrifuged at 4000 rpm and 4 °C for 5 min and resuspended in 5 ml of 5% sterile sugar. After two washes with 5% sugar, bacterial concentration was adjusted for each specific assay based on the optical density at 600 nm in a spectrophotometer (Pharmacia Biotech^®^, Ref. 80-2106-20). Spiked-sugar solutions were then offered to sand flies as later described (**Figure 1**).

**Figure 1:**
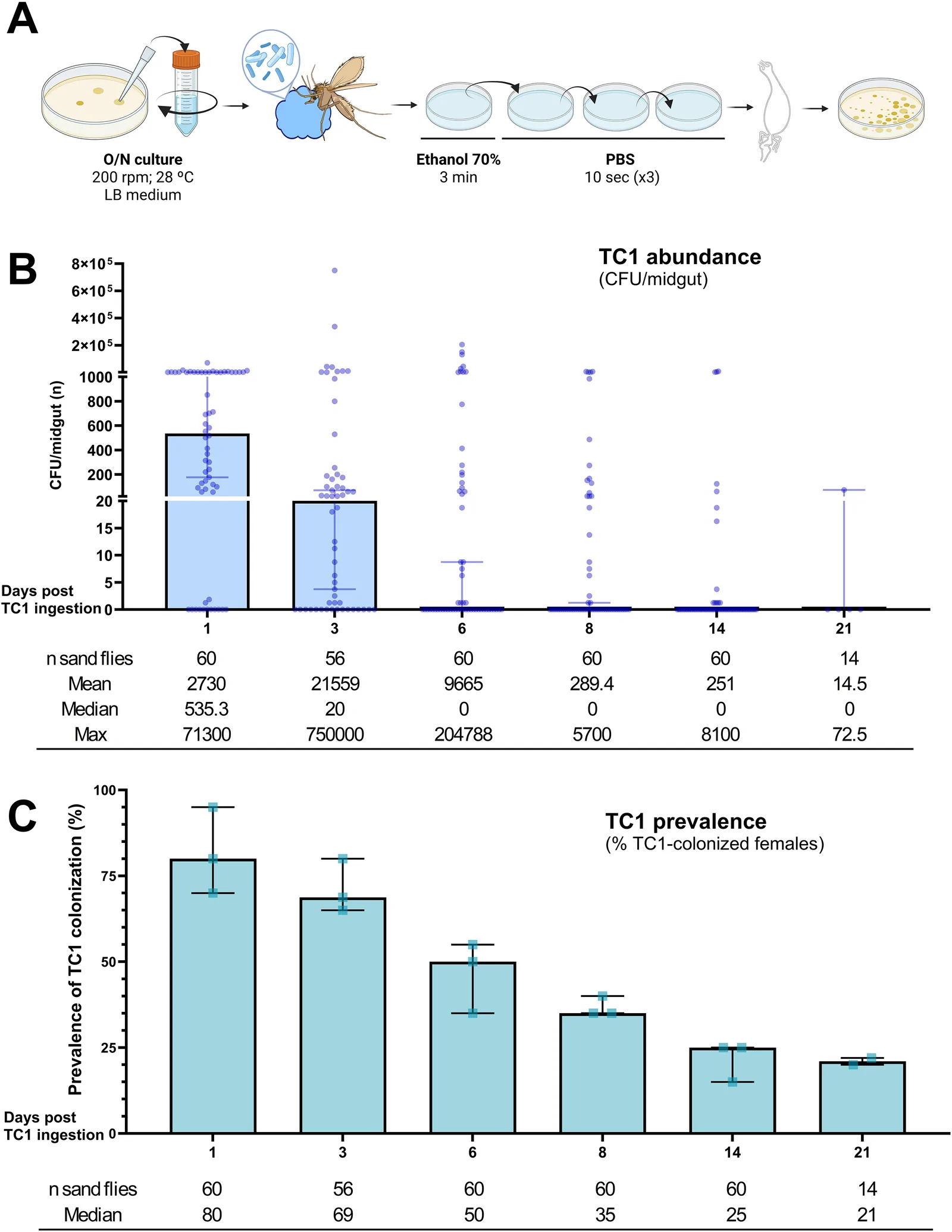
Temporal dynamics of TC1 midgut colonization and persistence in adult female *Phlebotomus perniciosus* (*Ppern*-BOA) following a TC1-containing sugar meal. **(A)** Experimental workflow. **(B, C).** TC1 colonization rate. Dots and bar-plots indicate median values with ±95% CI of median colony forming units (CFU) per single dissected female **(B)** or median TC1 prevalence per assay **(C)** per days post TC1 ingestion (X-axis). Data from 3 independent replicates are shown. Descriptive values are presented below each graph: dpsm, days post sugar meal; n assays, number of experiments included in the analysis; n sand flies, total number of individuals included in each analysis; Median, median values; nd, no data. Infographic created with BioRender.com.

### 2.4 Adult sand fly bacteria feeding

Newly emerged sand flies (1–3 days post-emergence) were offered sugar cotton balls soaked in a mixture of the final bacterium concentration (described below) and Brilliant Blue dye (400 ng/µl; Merck^®^, Ref. F80-717). One day after the sugar meal was offered (1 dpsm), female sand flies were systematically collected in low numbers using an entomological aspirator, anesthetized by exposure to CO_2_ (0.85 _bar_) for 5–10 sec, and transferred into a humid oviposition container (500 ml Nalgene plastic containers with a plaster-of-Paris base, commonly used for sand fly oviposition and colony maintenance) that had been previously frozen at -20 °C to extend anesthesia. Blue dye detection in sand flies was set as a proxy for sugar ingestion. Sugar-fed individuals were separated under a stereomicroscope with a pencil brush and deposited into a new colonization/infection cup. Counts of sugar-fed, unfed, and dead individuals were recorded (**Figure 2**). Sugar-fed sand flies were kept on a 30% sterile sugar diet.

**Figure 2:**
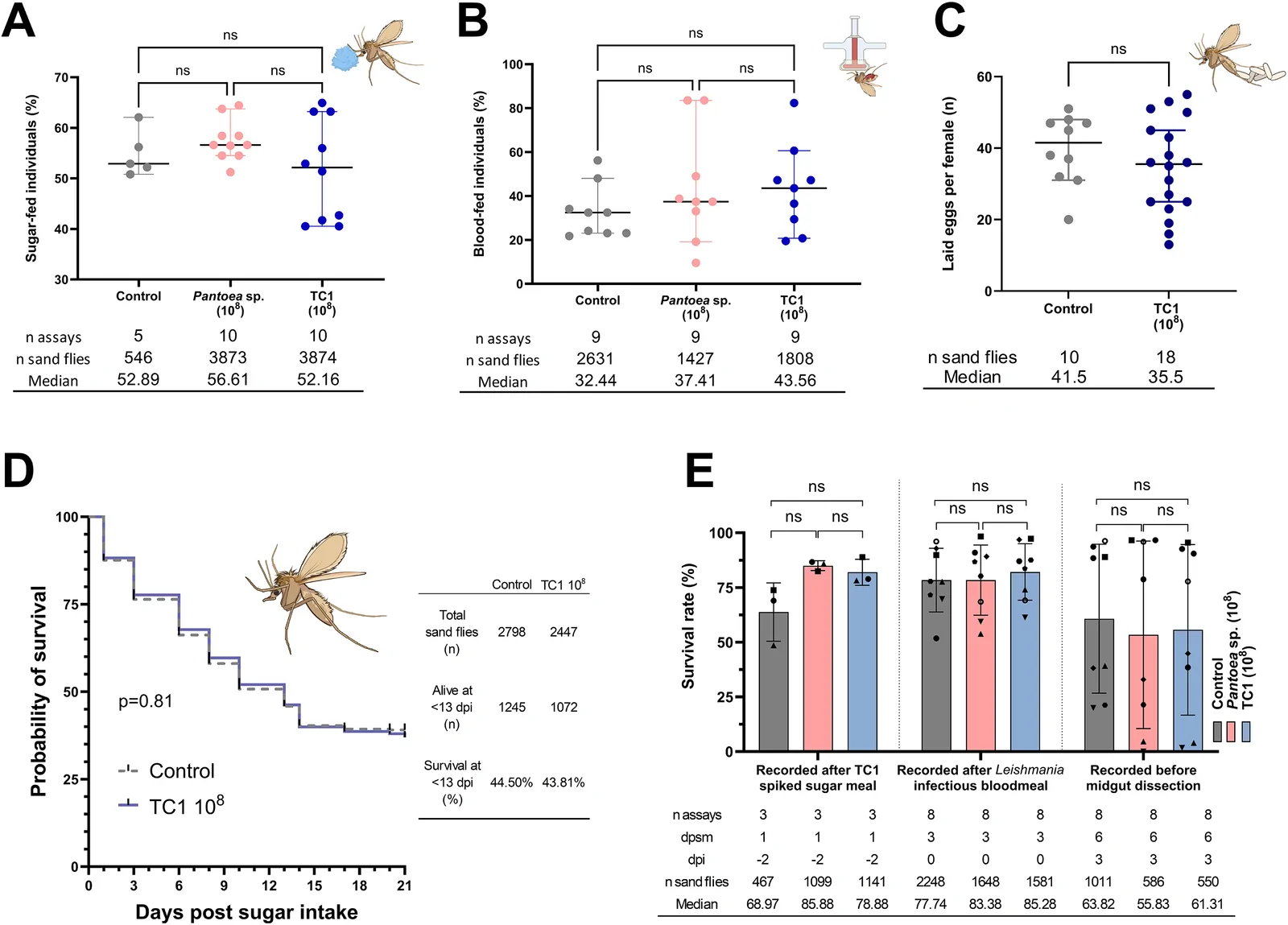
Effect of spiked-sugar feeding on female sand fly fitness. **(A-C)**. Dots and bar-plots indicate median values with ±95% CI of the estimated sugar feeding rate (Y-axis) of newly emerged female *Phlebotomus perniciosus* **(A)**, blood feeding rate at 3 days post-bacteria intake **(B)** or number of eggs laid per female **(C)** recorded during different assays. **(D)**. Probability of female sand fly survival (Y-axis) per day since spiked-sugar feeding (X-axis) from the total sand flies at the start of the experiment (Kaplan Meier test). **(E)**. Bars represent the mean percentage of sand fly survival per experiment (Y-axis) at key days post sugar meal (dpsm) or days post *Leishmania*-infection (dpi): ‘Recorded after TC1 spiked sugar meal’, ‘Recorded after *Leishmania* infectious bloodmeal’ and ‘Recorded before midgut dissection’. Gray, control sugar (no bacteria load); pink, *Pantoea dispersa* (10^8^ CFU/ml), and blue, TC1 (10^8^ CFU/ml). Dot shape represents paired data. Dunn *post-hoc* non-parametric tests **(A, B, E)** and non-parametric Mann-Whitney U test **(C)**. **(A-E)**. Descriptive variables are represented as a table below the graph: n assays: number of experiments included in the analysis; n sand flies: total number of individuals included in each analysis. ns: no statistical significance. Infographic created with BioRender.com.

### 2.5 TC1 colony quantification and molecular confirmation

Sand flies were collected, anesthetized by 5–10 sec exposure to CO_2,_ and transferred to a Petri dish containing 70% ethanol for 3 min for external cuticle sterilization. Individuals were then washed twice for 10 sec in PBS and kept in PBS until dissection, as described by Fraihi et al. (38). Dissections were conducted on a sterile slide using a stereomicroscope. After removing both wings and legs, the gut was extracted through the rear end of the abdomen. Foregut, hindgut, and Malpighian tubes were removed. Isolated midguts were then washed twice in two consecutive drops of PBS for 1–3 sec and transferred to 1.5 ml screw tubes containing 100 µl of LB medium and Benchmark zirconium 1 mm bulk beads (Merck^®^, Ref. BMSD113210TP). Samples were then homogenized in a mill homogenizer (Bead Ruptor Elite, Omni International) for 30 sec at 4 m/sec. Homogenized samples were then centrifuged for 1 min at 1500 rpm. Each homogenate sample was diluted in LB at two different concentrations (80/100; 1/100) for a final volume of 100 µl and was distributed on Amp/Gen LB agar plates using 4.5 mm spherical glass plating beads (Zymo Research^®^, Ref. S1001). Plates were incubated at 28 °C for 48 h, and colony-forming units (CFU) were recorded manually to enumerate viable bacteria within sand fly midguts. TC1 colonies were visually identified based on morphology. For each TC1-positive sample identified based on bacterial morphology, molecular confirmation was performed by sampling two independent single-colonies for each bacterial morphotype. Bacterial colonies were individually collected and resuspended in 10 µl of sterile water, which served as the template for colony PCR. Reactions were prepared using Hotsplit DNA polymerase (2.5 Units; Biotools^®^ B&M Labs, Ref. 10.562-4549), 5 µl of the corresponding reaction buffer, MgCl_2_ to a final concentration of 2 mM, dNTPs (Biotools^®^ B&M Labs) at a final concentration of 200 µM, specific primers for *Delftia* TC1 (D3F: 5’-CAGTAGGCGGTGATGTTCTT-3’ and D3R: 5’-GTTCAATCAGCCCTGCAATC-3’; Merck^®^) at a final concentration of 500 nM each, 4 µl of bacterial suspension and sterile distilled water to a final volume of 50 µl. PCR conditions consisted of an initial denaturation at 94 °C for 6 min, followed by 30 cycles of 30 s at 94 °C, 30 s at 57 °C, and 20 s at 72 °C, with a final extension at 72 °C for 10 min as previously described (34). Amplification products were separated on 1.5% agarose D1 low EEO gels (Condalab^®^, Ref. 8010), stained with Pronasafe Nucleic Acid Staining Solution (10 mg/ml; Condalab^®^), and visualized under UV illumination. Samples always consisted of individual midguts from single-dissected females. During each TC1-quantification assay, negative controls included 100 µl of PBS and LB used during dissections, as well as three independent dissected midguts of females collected from the laboratory colony. DNA extracted from a TC1 culture (180 ng) with a Speedtools Tissue DNA Extraction Kit (Biotools^®^ B&M Labs, Ref. 21.136-4195) was set as a positive control for colony PCR (**Figure 1**). A non-template control (NTC) with sterile water was also included during all PCR assays. Further, a preliminary molecular screening as here described for *Delftia* spp. was conducted before colonization and infection assays to confirm that this bacteria genus was not previously present in our sand fly colonies.

### 2.6 Colonization assays

Newly emerged sand flies (*Ppern*-BOA) were offered blue-dyed cotton balls (Brilliant Blue; 400 ng/µl) impregnated with 5% sugar solution spiked with 10^8^ CFU/ml of TC1 bacteria for 24 h. Sugar-fed individuals were later collected and kept on a sterile 30% sucrose diet. Twenty females were dissected per time point at 0, 1, 3, 5/6, 7/8, 9, 14/15, and 21-days post-sugar meal (dpsm). Dates were selected to cover early (0, 1 and 3), intermediate (5/6, 7/8 and 9), and late (14/15 and 21 days post sugar ingestion) stages of TC1 colonization and persistence within the sand fly midgut. Furthermore, day 6 also corresponded to the dissection timing used in the *in vivo *Leishmania** infection assays. TC1 abundance per midgut was quantified as previously described. Three independent biological replicates were conducted. To assess whether bacterial dose affected TC1 persistence in the sand fly midgut, an additional high-dose colonization assay was performed using the same experimental design but with 10^10^ CFU/ml TC1 in the sugar meal. In this assay, ten females were dissected per time point, and two independent biological replicates were performed. TC1 abundance and prevalence were compared between both bacterial doses (10^8^ and 10^10^ CFU/ml) using a non-parametric Mann-Whitney and Friedman tests for bacterial abundance and Odds Ratio (OR) for prevalence. Data was represented as bar diagrams.

### 2.7 *In vivo* anti-*L. infantum* effect evaluation assays of TC1 within *Ph. perniciosus*

Newly emerged sand flies were fed on a 5% sterile sucrose solution with no bacteria or spiked with either 10^8^ CFU/ml of *Pa. dispersa* or TC1. Sand flies were starved for 24 h before blood-feeding. Females were offered an infectious blood meal (10^6^ BOS1FL1 promastigotes/ml) at 2 dpsm (24 h after removing spiked sugar) through a chicken skin membrane in an entomological Rutledge-style glass feeder (40) (Pobel^®^, custom made) connected to a 37 °C water bath for 1 h. Defibrinated rabbit blood samples used during blood-feeding assays were obtained from Centro San Bernardo (CSB, Navarra, Spain) in accordance with European Directive 2010/63/EU. Females were systematically collected after blood feeding in low numbers with an entomological aspirator, anesthetized with a 5–10 sec exposure to CO_2_, and then sorted based on blood feeding status (non-blood-fed or fully blood-fed) on a humid and cold plaster of Paris container to extend insect anesthesia. Blood-fed females were separated into a new colonization/infection container until dissection at 5 days post *Leishmania*-infection (dpi), as previously described for TC1 quantification. Dissected midguts were collected into tubes containing 50 µl of LB and ruptured with a hand-held pestle tissue grinder (Thomas Scientific^®^, Ref. 3411D62) for 12 s. Homogenized midgut content was diluted for a final 0.05% formalin concentration (Thermo Scientific^®^, Ref. 15496105) to reduce parasite motility, and promastigote abundance was manually quantified in a Neubauer chamber (NanoEntek^®^, Ref. DHC-NO1) at 400x magnification. Promastigote quantification was conducted blindly and randomly (the study group was unknown to the counter, and samples were randomized before quantification) (**Figure 3**). Two preliminary assays and two major studies, each with a set of sand flies from independent *Ph. perniciosus* colonies (*Ppern*-BOA, and *Ppern*-FUE), were included. Median promastigote abundance was compared between the three sugar diets (no bacteria, *Pa. dispersa*-spiked or TC1-spiked sugar-fed females) through a non-parametric Kruskal-Wallis test and Dunn’s *post-hoc* tests. In one set of experiments, homogenized midguts (n=27) were plated for additional TC1 quantification to evaluate the correlation with parasite abundance. Spearman’s rank correlation test was conducted to evaluate the correlation between the log-transformed number of TC1 CFU and *Leishmania* promastigotes per sampled midgut. R^2^ and *p-values* are presented. For survival curves, dead sand flies were counted daily, while sugar and blood feeding rates were also recorded for all experiments. For the evaluation of the progression of the *Leishmania* infection in the sand fly midgut, two independent assays were conducted. Females fed on 10^10^ CFU/ml-TC1 spiked-sugar were offered an infectious blood meal (10^6^ BOS1FL1 promastigotes/ml) at 2 dpsm after 24 h of starvation. In this case, sand fly midguts were dissected 10 days after *L. infantum* infection (12 dpsm) as previously described and directly examined under a microscope at 100-400x magnification for a qualitative evaluation of the infection. Sand flies fed on sterile sugar (i.e., no bacteria) were used as control groups. Maturity of *Leishmania* infection was categorized blindly (study group was unknown to the observer) in line with Myskova et al. (41) and Martín-Martín et al. (39), as not infected individuals (no *Leishmania* promastigote detection), immature infections (promastigotes are not detected in high number in the stomodeal valve) or as a mature infection (high promastigote detection in the stomodeal valve). A Cochran-Armitage test was conducted to compare the *Leishmania*-infection maturity levels between TC1-fed sand flies and control individuals using the ‘CochranArmitageTest()’ function from the 'DescTools' package in RStudio (42) as a primary analysis. In addition, Fisher’s exact test was performed separately for each infection-level category as secondary descriptive analyses. Programmed R code is presented as **Supplementary Data 1**.

**Figure 3:**
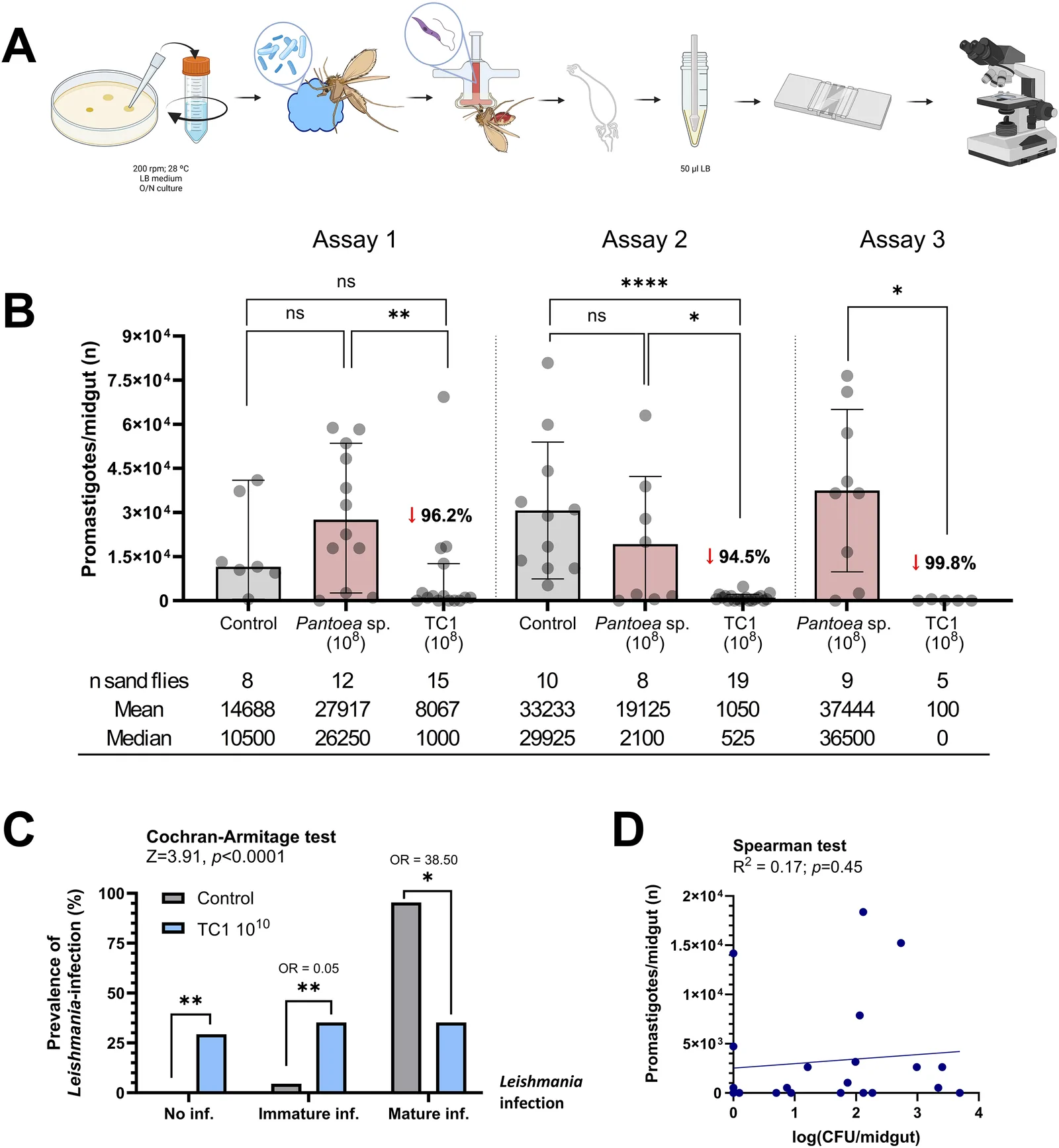
TC1 anti-*Leishmania infantum* (BOS1FL1) *in vivo* effect in *Phlebotomus perniciosus* (Ppern-BOA). **(A)**. Experimental workflow. **(B)**. Dots represent the median number of promastigotes per dissected midgut 5 days after an *L.* infantum infectious blood meal. Median and ±95% CI are presented as bar-plots. Data from three independent assays (i.e. biological replicates) is shown. Values were grouped per sugar diet: non-bacteria colonized sugar-feeding (gray, no-bacteria fed control), TC1-spiked (10^8^ CFU/ml, blue), and *Pantoea dispersa*-spiked (10^8^ CFU/ml, pink, bacteria-fed control). Non-parametric Kruskal Wallis test followed by Dunn´s *post hoc* test are presented above the bars (ns, non-significant; *, *p*< 0.05; ****, *p*< 0.0001). **(C)**. Bars represent the percentage of infected females based on infection level. Sand flies were classified as not infected (no promastigotes detected in midgut), immaturely infected (promastigotes are detected in midgut but stomodeal valve is not highly infected) or maturely infected (stomodeal valve is highly colonized by promastigotes). Data from two independent experiments are presented and was grouped by sugar diet: non-bacteria colonized sugar-feeding (gray, control) or TC1-spiked (10^8^ CFU/ml, blue). **(D)**. Dots represent *Leishmania*-promastigote count (Y-axis) per logarithmic TC1 CFU count (X-axis) per dissected female midgut after TC1 midgut colonization (10^10^ CFU/ml). The blue line represents a linear fit shown for visualization purposes. No statistical correlation was assumed (n = 27 sand flies; Spearman R^2^ = 0.17, *p*= 0.45). Data from an independent experiment. Infographic created with BioRender.com. #Fisher's exact test and Odds Ratio (OR) comparing the prevalence per infection maturity level between control and TC1-fed sand flies are presented over bars (**, *p*< 0.001; ***, *p*< 0.0001).

### 2.8 Bacteria supernatant preparation

Overnight 5 ml cultures of TC1 and *Pa. dispersa* were centrifuged at 4000 rpm for 5 min at 4 °C. The obtained pellets were resuspended in 5 ml of M9 (Merck^®^, Ref. M6030) for *in vivo* experiments or enriched RPMI medium for *in vitro* assays, as described in the diagram on **Figure 4A**. After a 2 h culture at 200 rpm at 28 °C, cultures were centrifuged, and supernatants were collected, filter sterilized (<0.22 µm), and stored at -80 °C until use (**Figure 4**). A 2 h incubation period was used, as optimization studies (unpublished data) demonstrated that this duration was sufficient to achieve effective harmane concentrations in TC1 cell-free supernatants.

**Figure 4:**
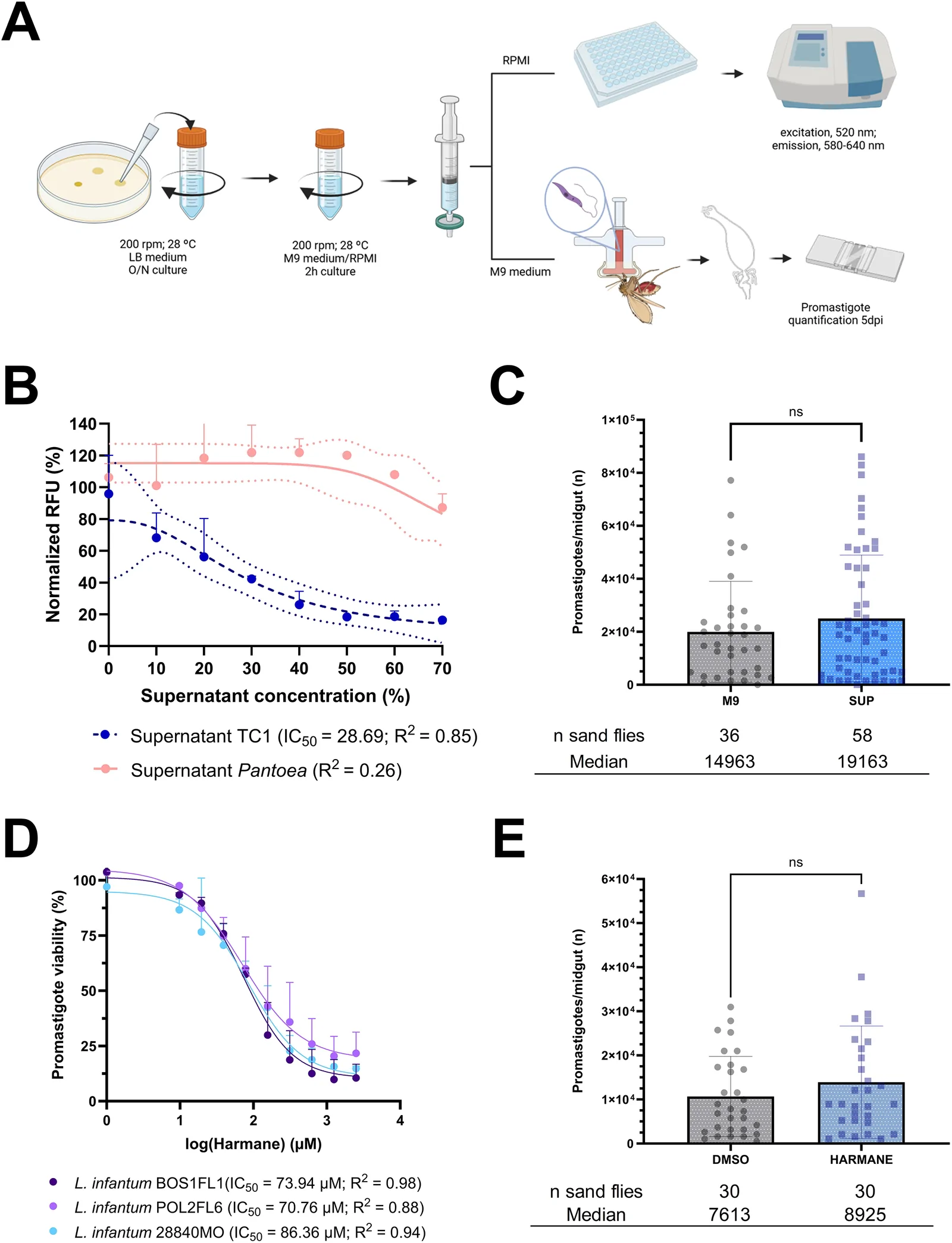
Anti-*Leishmania infantum* effect of TC1-supernatant **(A-C)** and harmane **(D, E)**. **(A)**. TC1-supernatant experimental workflow. **(B)**. Bacteria supernatant *in vitro* assay 48 h after plate setting. Each point represents the recorded normalized fluorescence value (RFU (%)) for a serial dilution of bacteria supernatant (blue, TC1; pink, *Pantoea dispersa*). **(C)**. TC1-supernatant effect on promastigote abundance per female midgut. Each point represents the amount of *L. infantum* promastigotes per midgut for each independent dissected female 5 days after an infectious blood meal (10^6^ *L. infantum* promastigotes/ml of defibrinated rabbit blood) spiked for a final concentration of 50% of M9-media (negative control, gray) or TC1-supernatant (blue). **(B, C)**. Bars represent medians with 95% confidence intervals. P values from Mann-Whitney U tests are indicated above the bar plots; ns, not significant. **(D)**. Harmane *in vitro* promastigote viability assay 48 h after plate setting. Each point represents the mean value of percentual promastigote viability (Y-axis) per logarithmic drug concentration (X-axis). Each color represents the obtained values for each of the three evaluated *L. infantum* strains. IC_50,_ R^2^, non-linear fit curves, and medians ± 95% CI are shown for each strain. **(E)**. Harmane *in vivo* promastigote viability 5 days post-spiked infectious blood meal. Points represent the amount of *L. infantum* promastigotes per midgut for each independent dissected female 5 days after an infectious blood meal (10^6^ *L. infantum* promastigotes/ml of defibrinated rabbit blood) spiked for a final concentration of 1% DMSO (negative control) or 100 µM of harmane. Infographic created with BioRender.com.

### 2.9 *In vivo* anti-*L. infantum* effect evaluation assays of TC1 derivatives within *Ph. perniciosus*

TC1 derivatives anti-*L. infantum* effect within *Ph. perniciosus* (*Ppern*-BOA) were also evaluated. Female sand flies, after 24 h of starvation, were offered an infectious blood meal (10^6^ BOS1FL1 promastigotes/ml) that contained 50% of TC1-supernatant or M9-medium as a control group. In another set of experiments, infectious blood meals were prepared with 100 µM harmane (Merck^®^, Ref. 103276) or 1% DMSO (Merck^®^, Ref. D8418) as a negative control, as it is the harmane solvent. Female midguts were dissected at 5 dpi for *L. infantum* promastigote quantification as previously described (**Figure 4**). Three replicates were conducted for each experimental assay. For each set of experiments, median promastigote abundance between study and control groups were compared through a non-parametric Mann-Whitney U test. Dead sand flies count per date, and sugar and blood feeding rates were also recorded.

### 2.10 Evaluation of TC1 and TC1 derivatives on the fitness of *Ph. perniciosus*

To assess the potential fitness cost associated with TC1 colonization, sand fly survival was monitored and compared across experimental groups (non-bacteria fed and previously TC1-fed sand flies) using a Kaplan-Meier analysis and the log-rank Mantel-Cox test. Additionally, daily mortality after TC1 *in vivo* anti-*Leishmania* assays (Methods section 2.7) was registered and compared among study groups at certain dates during experiments (one day post-sugar meal or dpsm, right after O/N sugar feeding; 2 dpsm, right after a *L. infantum* infectious blood meal; 6 dpsm, before dissecting the midgut of *L. infantum* infected sand flies) using a Friedman and Dunn’s multiple comparisons tests. Spiked-sugar and blood-feeding rates among sugar meal diets were compared across groups using a non-parametric Kruskal-Wallis test. To assess fecundity, hamster blood-fed *Ph. perniciosus* females (*Ppern*-BOA) were offered a sugar meal containing TC1 bacteria at 10^8^ CFU/ml. Blood-fed sand flies used in this study were obtained from surplus individuals generated during routine colony maintenance, which involved feeding on anesthetized hamsters under animal study protocol PROEX 332.0/25 authorized by the Bioethics and Animal Welfare Committee of Madrid, Spain; no animals were used specifically for this work. Sugar-fed females were then individually maintained in glass vials under 100% humidity with access to a sterile 30% sucrose diet. The number of eggs laid per female was counted and compared between TC1-fed and bacteria-free control females using a non-parametric Mann-Whitney U test.

Survival rate was compared between TC1-supernatant and M9, as well as harmane and DMSO fed females through independent Kaplan-Meier tests based on daily mortality. Blood-feeding rate was also compared through non-parametric Mann-Whitney U tests and represented as bar diagrams.

### 2.11 *In vitro* anti-*L. infantum* effect of TC1 derivatives

To assess whether secreted bacterial metabolites could be responsible for the anti-*Leishmania* activity observed with live TC1, TC1 cell-free supernatant and harmane, a β-carboline alkaloid secreted by TC1 and previously associated with anti-*Leishmania* activity, were tested *in vitro* against *L. infantum* promastigotes. Dose response curves of harmane, DMSO (as a negative control of harmane), and the anti-*Leishmania* spp. drug antimony potassium tartrate (Sb^3+^; VWR^®^, Ref. 1.08092.0250), were determined against the three strains of *L. infantum* promastigotes (BOS1FL1, POL2FL6, and 28840MO). Harmane was first evaluated against BOS1FL1, the main strain used in the *in vivo* sand fly infection assays. After detecting an anti-parasitic effect, the two other additional *L. infantum* strains were included in the study. These additional strains were included to assess the consistency of the *in vitro* effect across clinically and epidemiologically relevant parasite isolates. Serial 1/2 dilutions (n=10) were prepared in concentrations ranging from 2500 to 0 μM for harmane and DMSO, and 4800 to 0 μM for Sb^3+^. Dilutions were prepared in technical triplicates and added to flat-bottomed 96-well cell culture microtiter plates (Falcon^®^, Ref. 351172) containing 10^6^ parasites per well. Marginal wells were filled with distilled water to avoid the edge effect. After 24 h, 20 µL (10% v/v) of AlamarBlue Cell Viability Assay Reagent (Biosciences^®^, Ref. 786-923) were added to each well, and plates were protected from light exposure and incubated at 27 °C. Fluorescence readings were recorded at 8, 24, 48, and 72 h post addition of AlamarBlue reagent (excitation, 520 nm; emission, 580–640 nm) using a plate reader (GloMax Explorer, Promega^®^). Three independent biological replicates were performed for each *L. infantum* strain. Promastigote viability was calculated for each assay as the percentual difference between the recorded absorbance and the average absorbance of the corresponding DMSO control. Average promastigote viability values were obtained from technical replicates for each drug concentration and fluorescence reading time point. Non-linear regression analyses were performed using a log(agonist) versus response – variable slope (four-parameter logistic) model (4PL) to assess the relationship between drug concentration and promastigote viability. Data from the three independent biological replicates were grouped to determine the IC_50_ values of harmane for each *L. infantum* strain.

Additionally, a single assay with a technical triplicate was conducted to evaluate the *in vitro* effect of TC1 and *Pa. dispersa* supernatant on the BOS1FL1 *L. infantum* strain. Supernatant was prepared in enriched RPMI medium as previously described. Concentrations of 0, 10, 20, 30, 40, 50, 60, and 70% of bacterial supernatants were prepared in enriched RPMI media and added to a 96-well plate containing 10^6^ parasites per well in triplicate. Similar conditions, fluorescence readings and data handling were conducted as previously explained.

### 2.12 Data analysis

Data obtained from different replicates per experiment were grouped for statistical analyses when specified. Descriptive parameters were obtained per experiment (n, median, mean, min, max, and/or sd). Data cleaning and handling were conducted in Microsoft Excel (Windows version Microsoft Office Professional Plus 2019; Microsoft Corporation), while data representation and descriptive and inferential analyses were performed in GraphPad Prism (Windows version 8.0.2 (263); GraphPad Software, San Diego, CA, USA) and RStudio (Windows version 4.5.2; The R Foundation for Statistical Computing). Illustrations were created with the assistance of BioArt (BioArt-Public Domin, National Institute of Health), BioRender (^©^BioRender) and Microsoft Publisher (Windows version Microsoft Office Professional Plus 2019; Microsoft Corporation).

## 3 Results

### 3.1 TC1 midgut persistence

#### 3.1.1 TC1 colonizes and persists in the midgut of *Ph. perniciosus*

*Delftia tsuruhatensis* TC1 remained detectable for up to 21 days in surviving flies, consistent with prolonged persistence in the sand fly gut. TC1 prevalence and median abundance peaked at 1 dpsm, reaching approximately 500 CFU/midgut, and declined thereafter (**Figure 1**). After 3 dpsm, most positive sand flies carried low bacterial loads (≤20 CFU/midgut), although occasional individuals remained highly colonized, with loads ranging from 10³ to 10^5^ CFU/midgut (**Figure 1B**). To evaluate the effect of the initial bacterial dose on colonization, two TC1 concentrations were tested in sugar meals. Although median TC1 abundance at 1 dpsm did not differ between the 10^8^ and 10^10^ CFU/ml treatments, TC1 prevalence was higher in flies receiving 10^8^ CFU/ml on days 5 and 8 (**Supplementary Figure 1**). Therefore, 10^8^ CFU/ml was selected for all subsequent experiments. Results from individual assays are shown in **Supplementary Figure 2**.

#### 3.1.2 TC1 does not affect the fitness of *Ph. perniciosus*

Feeding sand flies on TC1-containing sugar (10^8^ CFU/ml) did not affect subsequent sugar or blood-feeding rates at 3 dpsm (**Figures 2A, B**). No difference in the number of eggs laid per female (fecundity) was observed between control and TC1-fed sand flies 7 days after a non-infectious blood meal (**Figure 2C**). Additionally, bacteria ingestion did not cause an increase in mortality (**Figure 2D**). Bacteria-fed sand flies showed a similar mortality rate to the control group at 0 or 3 days after an *L. infantum*-infected blood meal (**Figure 2E**). Furthermore, TC1-derivatives did not cause any effect on sand fly mortality during the *in vivo* assays (**Supplementary Figure 3**).

### 3.2 TC1 and its derivatives exhibit anti-*L. infantum* activity

#### 3.2.1 TC1 presents an anti-*L. infantum* effect within phlebotomine sand flies

Results from two independent preliminary assays were subsequently confirmed (**Supplementary Figures 4A, B**) in three independent assays with TC1-colonized *Ph. perniciosus* (10^8^ CFU/ml; *Ppern*-BOA) (**Figure 3A**) which showed significant reductions of promastigote counts (BOS1FL1) ranging from 90.5 - 98.2% compared to control sand flies (fed on sugar without bacteria)); and 75 - 99.7% reduction when compared to those fed on *Pa. dispersa* spiked-sugar (**Figure 3B**). Furthermore, similar results were observed with a different *Ph. perniciosus* colony (*Ppern*-FUE) during an independent assay for both non-fed with bacteria (95.2% promastigote count reduction) and *Pa. dispersa* fed sand flies (96.5% reduction) (**Supplementary Figure 4C**). Additionally, two independent experiments were performed to evaluate the *L. infantum* (BOS1FL1) infection maturity in sand flies. Mature *Leishmania* infections in the sand fly vector are characterized by the presence of metacyclic promastigotes colonizing the stomodeal valve, a prerequisite for sand fly infectivity and subsequent parasite transmission (41). A lineal negative tendency between infection maturity and TC1-ingestion was observed through a Cochran-Armitage test. Prior colonization with TC1 (10^10^ CFU/ml) reduced the proportion of sand flies infected with *L. infantum* (Fisher’s exact test: *p* < 0.005*;* OR = 0.05 [0.004-0.47]). Furthermore, infected TC1-colonized females were more likely to harbor immature midgut infections than control individuals (Fisher’s exact test: *p* < 0.0001*;* OR = 38.50 [5.08-428.8], **Figure 3C**). To understand whether the anti-*Leishmania* effect was associated with the degree of TC1 colonization, an additional infection experiment was performed in which both bacterial and parasite loads were quantified in individual midguts. No significant correlation between total TC1 CFU and *L. infantum* BOS1FL1 promastigote counts per midgut was observed (Spearman R^2^ = 0.17, *p* = 0.45) (**Figure 3D**).

#### 3.2.2 TC1-derivatives show *in vitro* dose dependent anti-*L. infantum* promastigote effect

To understand if secreted metabolites were potentially responsible for the anti-*Leishmania* activity of TC1, its cell free supernatant and harmane, a β-carboline alkaloid secreted by TC1, were screened *in vitro* against *L. infantum* promastigotes. We tested increasing concentrations of TC1-supernatant which showed an *in vitro* anti-*L. infantum* effect against BOS1FL1. In this regard, some reduction was observed at the lowest tested TC1-supernatant concentration (10%). The highest evaluated concentration (70%) showed a promastigote reduction ranging from 82.78 to 83.82% (mean 83.41%) (**Figure 4B**). A single experiment with three technical replicates was conducted. Afterwards, harmane also showed anti-*L. infantum* effect against three independent viscerotropic *Leishmania* strains, with IC_50_ values ranging between 70.76 and 86.36 µM at 48 h post plate-setting (BOS1FL1, IC_50_ = 73.94 µM; POL2FL6, IC_50_ = 70.76 µM, and 28840MO, IC_50_ = 86.36 µM) (**Figure 4D**). Similar results were obtained among biological replicates and with Sb^+3^, a well-characterized anti-*Leishmania* spp. drug commonly used for treating VL (3) (**Supplementary Table 1**). Interestingly, when added to an *L. infantum* infectious blood meal, neither harmane nor TC1-supernatant showed an inhibitory effect within the midgut of phlebotomine sand flies (**Figures 4C**, **E**).

## 4 Discussion

### 4.1 TC1 establishes persistent gut colonization and impairs *L. infantum* development in *Ph. perniciosus*

Despite advances in diagnosis and treatment, VL continues to pose a substantial public health burden, while urbanization, climate change, and the expansion of vector habitats are increasing transmission risk in previously unaffected settings (2). Consequently, the development of novel transmission-blocking strategies that complement existing control measures is urgently needed (43). TC1 was originally isolated from insectary mosquitoes at GSK and has been shown to successfully colonize *Anopheles* mosquitoes, where it exerts anti-*Plasmodium* activity through the secretion of harmane (34). Subsequently, Cecilio et al. (29) demonstrated that TC1 colonizes *Ph. duboscqi* sand flies and interrupts transmission of **Leishmania* major*, the causative agent of CL, proposing that this effect is mediated indirectly through TC1-induced gut dysbiosis. The objective of the present study was to determine whether the utility of TC1 could be extended beyond CL to VL using *Ph. perniciosus*, the predominant vector of *L. infantum* in Spain and across much of the Mediterranean basin.

A critical prerequisite for the development of TC1 as a microbiota-based intervention is its ability to be effectively acquired by sand flies and to establish stable colonization within the gut. Our results demonstrate that TC1 can be effectively introduced into *Ph. perniciosus* through sugar-soaked cotton feeds and subsequently persist within the sand fly midgut for at least 21 days post-ingestion. Notably, a single exposure to a TC1-supplemented sugar meal was sufficient to achieve detectable colonization throughout the study period, indicating that TC1 can stably persist in the sand fly gut over an extended duration. Importantly, TC1 carriage was associated with a marked reduction in *L. infantum* promastigote abundance during the early stages of infection (5 dpi) compared with control flies. Although direct transmission experiments were beyond the scope of this study, we consistently observed a substantial reduction in stomodeal valve infections in TC1-carrying sand flies across replicate experiments. Colonization of the stomodeal valve is a critical prerequisite for successful parasite transmission and has been considered a more relevant predictor of transmission competence than overall midgut parasite burden (41). Together, the qualitative and quantitative reduction in *L. infantum* infection is consistent with a reduction in the transmission potential of infected *Ph. perniciosus*. While further studies using animal transmission models are required to directly validate this hypothesis, our findings, together with previous work demonstrating transmission interruption by TC1 in CL (29), support the continued evaluation of TC1-based approaches to interrupt *Leishmania* transmission.

Importantly, our work represents the first demonstration that TC1 can impair the development of *L. infantum* within *Ph. perniciosus*, the principal vector of VL in the Mediterranean region. These findings substantially expand previous observations in CL (29) and indicate that the anti-*Leishmania* activity of TC1 is not restricted to a single parasite species, disease manifestation, or sand fly vector. Together with previous studies (29, 34), the findings presented here support the potential of TC1 as a versatile microbiota-based platform for the control of vector-borne pathogens.

### 4.2 Possible mechanisms underlying the anti-*Leishmania* effect

Understanding the complex vector–parasite–microbiota interactions governing pathogen establishment and transmission is fundamental for the development of microbiota-based control strategies (35, 44). To investigate possible mechanisms underlying the observed phenotype we evaluated the activity of TC1-derived products against *L. infantum* promastigotes both *in vitro* and *in vivo*. Harmane, the β-carboline alkaloid previously identified as a bioactive metabolite of TC1 (34), demonstrated significant dose-dependent anti-parasitic activity *in vitro*. Additionally, similar results were observed with cell-free TC1 supernatant, although only a single replicate was performed. These preliminary findings suggest that secreted bacterial metabolites can contribute to parasite inhibition.

Interestingly, this phenotype could not be reproduced *in vivo* when either TC1-supernatant or harmane was incorporated into infectious blood meals. Several factors could contribute to this discrepancy. Under *in vitro* conditions, parasites were continuously exposed to harmane and supernatant, whereas in the sand fly gut, exposure is likely influenced by digestion dynamics, parasite developmental stage, and local concentrations achieved following blood feeding. Consequently, the temporal and spatial interactions required to reproduce the antiparasitic effects observed *in vitro* may not be achieved under experimental *in vivo* conditions used here. In addition, the lack of correlation between TC1 abundance and promastigote burden suggests that parasite inhibition cannot be explained by bacterial abundance. Whether the observed effect is mediated by highly potent secreted metabolites, as previously described for TC1-mediated suppression of *Plasmodium* in mosquitoes (34), or by the interaction with the resident microbiota (29), or by the combination of both factors, remains unknown.

Although mechanistic studies were beyond the scope of the current work and represent a limitation of the study, future investigations integrating metabolite quantification and microbiota profiling within the sand fly gut will provide valuable insights into the processes underlying the observed phenotype. Such studies should focus on quantifying the temporal dynamics of harmane production in the sand fly gut, identifying additional bioactive metabolites, and characterizing interactions between TC1 and the resident microbiota that may contribute to parasite suppression, particularly given that both blood feeding and *Leishmania* infection are known to alter gut bacterial communities (27, 35, 45).

### 4.3 Translational potential and delivery strategies for TC1

Our findings demonstrate that TC1 can be efficiently introduced into laboratory-reared adult sand flies through bacteria-supplemented sugar meals and subsequently establish a persistent gut colonization. The translation of this delivery approach to field settings now represents an important next step. Bacterial delivery through sugar feeding has previously shown promise for TC1 and other symbiotic bacteria in sand flies (29, 30, 32), supporting the feasibility of this approach under operational conditions.

Existing attractive targeted sugar bait (ATSB)-based interventions have demonstrated efficacy against sand flies (46), although concerns regarding the ecological impact of insecticide-based active ingredients on target and non-target organisms may limit widespread implementation. In this context, TC1 offers an attractive alternative biological intervention. Notably, TC1-fed *Ph. perniciosus* exhibited no detectable fitness costs, even following an infectious blood meal. This contrasts with observations in *Ph. duboscqi*, where increased mortality was reported in *L. major*-infected flies carrying TC1 (29). Such differences suggest that the fitness consequences associated with TC1 may depend on the specific vector–parasite–bacterium interaction. Further studies employing different sand fly and *Leishmania* species combinations and/or experimental conditions will be necessary to better understand these effects and their implications for the development and deployment of TC1-based interventions.

Successful field implementation will also require a deeper understanding of sand fly sugar-feeding ecology. As reported for mosquitoes (47), identifying natural sugar sources exploited by sand flies will be essential for recognizing ecological factors that could influence TC1 uptake. This is particularly relevant because ingested sugars are predominantly directed to the crop rather than the midgut, whereas blood meals are delivered directly into the midgut (48, 49). Optimization of bait formulations, including adjustment of sucrose concentration and incorporation of phagostimulants (50), may therefore enhance bacterial uptake and improve the likelihood of successful gut colonization. Additionally, alternative routes of bacterial introduction as the supplementation of larval breeding substrates (51) deserve further investigation as strategies to improve dissemination and field applicability.

Further studies need to clarify the mode of action of TC1 and, most importantly, to determine whether the reductions in parasite burden and stomodeal valve colonization observed here translate into reduced transmission of *L. infantum*. Nevertheless, the results presented identify TC1 as a promising candidate for the environmentally sustainable biological control of VL. Its persistence within the vector midgut, together with its ability to significantly reduce *L. infantum* abundance and impair parasite colonization of the stomodeal valve, supports its further evaluation as a novel microbiota-based intervention for the control of leishmaniosis transmission in endemic settings.

## Data Availability

The raw data supporting the conclusions of this article will be made available by the authors, without undue reservation.

## References

[1] Akhoundi M Kuhls K Cannet A Votýpka J Marty P Delaunay P . A historical overview of the classification, evolution, and dispersion of *Leishmania* parasites and sandflies. PloS NeglTrop Dis. (2016) 10:e0004349. doi: 10.1371/journal.pntd.0004349 26937644 PMC4777430

[2] Maia C . Sand fly-borne diseases in Europe: epidemiological overview and potential triggers for their emergence and re-emergence. J Comp Pathol. (2024) 209:6–12. doi: 10.1016/j.jcpa.2024.01.001 38320331

[3] Pareyn M Alves F Burza S Chakravarty J Alvar J Diro E . The leishmaniasis. Nat Rev Dis Primers. (2025) 11. doi: 10.1038/s41572-025-00663-w 41266459

[4] Soong L Henard CA Melby PC . Immunopathogenesis of non-healing American cutaneous leishmaniasis and progressive visceral leishmaniasis. Semin Immunopathol. (2012) 34:735–51. doi: 10.1007/s00281-012-0350-8 23053396 PMC4111229

[5] Wamai RG Kahn J Mcgloin J Ziaggi G . Visceral leishmaniasis: a global overview. J Glob Health Sci. (2020) 2. doi: 10.35500/jghs.2020.2.e3

[6] WHO . Leishmaniosis (2023). Available online at: https://www.who.int/es/news-room/fact-sheets/detail/leishmaniasis (Accessed July 16, 2026).

[7] Hotez PJ . Southern Europe’s coming plagues: vector-borne neglected tropical diseases. PloS NeglTrop Dis. (2016) 10:e0004243. doi: 10.1371/journal.pntd.0004243 27362640 PMC4928793

[8] Alvar J Cañavate C Gutiérrez-Solar B Jiménez M Laguna F López-Vélez R . *Leishmania* and human immunodeficiency virus coinfection: the first 10 years. Clin Microbiol Rev. (1997) 10:298–319. doi: 10.1128/cmr.10.2.298 9105756 PMC172921

[9] Moore E Lockwood D . Treatment of visceral leishmaniasis. J Glob Infect Dis. (2010) 2:151–58. doi: 10.4103/0974-777X.62883 PMC2889655 20606971

[10] Gillespie PM Beaumier CM Strych U Hayward T Hotez PJ Bottazzi ME . Status of vaccine research and development of vaccines for leishmaniasis. Vaccine. (2016) 34:2992–95. doi: 10.1016/j.vaccine.2015.12.071 26973063

[11] De Freitas Milagres T López-De-Felipe M Da Silva WJ Martín-Martín I Gálvez R Da Silva OS . Same parasite, different outcomes: unraveling the epidemiology of **Leishmania* infantum* infection in Brazil and Spain. Trends Parasitol. (2023) 39:774–85. doi: 10.1016/j.pt.2023.06.008 37442747

[12] Dantas-Torres F . The role of dogs as reservoirs of *Leishmania* parasites, with emphasis on *Leishmania* (*Leishmania*) *infantum* and *Leishmania* (*Viannia*) *braziliensis*. Vet Parasitol. (2007) 149:139–46. doi: 10.1016/j.vetpar.2007.07.007 17703890

[13] Jiménez M González E Martín-Martín I Hernández S Molina R . Could wild rabbits (*Oryctolagus cuniculus*) be reservoirs for **Leishmania* infantum* in the focus of Madrid, Spain? Vet Parasitol. (2014) 202:296–300. doi: 10.1016/j.vetpar.2014.03.027 24774435

[14] Molina R Jiménez M Cruz I Iriso A Martín-Martín I Sevillano O . The hare (*Lepus granatensis*) as potential sylvatic reservoir of **Leishmania* infantum* in Spain. Vet Parasitol. (2012) 190:268–71. doi: 10.1016/j.vetpar.2012.05.006 22677135

[15] Alvar J Gutiérrez-Solar B Pachón I Calbacho E Ramírez M Vallés R . AIDS and **Leishmania* infantum*. New approaches for a new epidemiological problem. Clin Dermatol. (1996) 14:541–46. doi: 10.1016/0738-081X(96)00046-6 8889333

[16] Ready PD . Biology of phlebotomine sand flies as vectors of disease agents. Annu Rev Entomol. (2013) 58:227–50. doi: 10.1146/annurev-ento-120811-153557 23317043

[17] González E Jiménez M Hernández S Martín-Martín I Molina R . Phlebotomine sand fly survey in the focus of leishmaniasis in Madrid, Spain, (2012–2014): seasonal dynamics, **Leishmania* infantum* infection rates and blood meal preferences. Parasit Vectors. (2017) 10:368. doi: 10.1186/s13071-017-2309-z PMC5540423 28764772

[18] Risueño J Verdú-Serrano E Alessandra M Pérez-Cutillas P Molina R Jimenez M . Revisiting the largest human leishmaniasis outbreak in Western Europe (Madrid, Spain): a follow-up entomological study in 2023 and 2024. ParasitVectors. (2026) 19:222. doi: 10.1186/s13071-026-07336-x PMC13185400 41965749

[19] Kamhawi S . The yin and yang of leishmaniasis control. PloS NeglTrop Dis. (2017) 11:e0005529. doi: 10.1371/journal.pntd.0005529 28426716 PMC5398490

[20] Alexander B Maroli M . Control of phlebotomine sandflies. Med Vet Entomol. (2003) 17:1–18. doi: 10.1046/j.1365-2915.2003.00420.x 12680919

[21] Bezerra-Santos MA Dantas-Torres F Maia C Volf P Otranto D . Bio-ecology and management of phlebotomine sand flies: unraveling the complexity of vector control. J Pest Sci. (2026) 99:11. doi: 10.1007/s10340-025-01992-1

[22] Balaska S Fotakis EA Chaskopoulou A Vontas J . Chemical control and insecticide resistance status of sand fly vectors worldwide. PloS NeglTrop Dis. (2021) 15:e0009586. doi: 10.1371/journal.pntd.0009586 34383751 PMC8360369

[23] Karunaratne S Surendran S . Mosquito control: A review on the past, present and future strategies. J Natl Sci Found Sri Lanka. (2022) 50. doi: 10.4038/jnsfsr.v50i0.11244

[24] Becker N . Bacterial control of vector-mosquitoes and black flies. In: Entomopathogenic Bacteria: From Laboratory to Field Application. Dordrecht: Springer (2000) 383–98.

[25] Hurwitz I Hillesland H Fieck A Das P Durvasula R . The paratransgenic sand fly: a platform for control of *Leishmania* transmission. Parasit Vectors. (2011) 4:82. doi: 10.1186/1756-3305-4-82 21595907 PMC3121692

[26] Karimian F Vatandoost H Rassi Y Maleki-Ravasan N Mohebali M Shirazi MH . Aerobic midgut microbiota of sand fly vectors of zoonotic visceral leishmaniasis from northern Iran, a step toward finding potential paratransgenic candidates. Parasit Vectors. (2019) 12:10. doi: 10.1186/s13071-018-3273-y 30616668 PMC6322272

[27] Kelly PH Bahr SM Serafim TD Ajami NJ Petrosino JF Meneses C . The gut microbiome of the vector *Lutzomyia longipalpis* is essential for survival of **Leishmania* infantum*. MBio. (2017) 8:10–1128. doi: 10.1128/mbio.01121-16 PMC5241394 28096483

[28] Louradour I Monteiro CC Inbar E Ghosh K Merkhofer R Lawyer P . The midgut microbiota plays an essential role in sand fly vector competence for *Leishmania* major. Cell Microbiol. (2017) 19:e12755. doi: 10.1111/cmi.12755 28580630 PMC5587349

[29] Cecilio P Rogerio LA D. Serafim T Tang K Willen L Iniguez E . *Leishmania* sand fly-transmission is disrupted by *Delftia tsuruhatensis* TC1 bacteria. Nat Commun. (2025) 16:3571. doi: 10.1038/s41467-025-58769-4 40341020 PMC12062286

[30] Sant'Anna MR Diaz-Albiter H Aguiar-Martins K Al Salem WS Cavalcante RR Dillon VM . Colonisation resistance in the sand fly gut: *Leishmania* protects *Lutzomyia longipalpis* from bacterial infection. Parasit Vectors. (2014) 7:329. doi: 10.1186/1756-3305-7-329 PMC4112039 25051919

[31] Vaselek S Sarac BE Uzunkaya AD Yilmaz A Karaaslan C Alten B . Identification of *Ochrobactrum* as a bacteria with transstadial transmission and potential for application in paratransgenic control of leishmaniasis. Parasitol Res. (2024) 123:82. doi: 10.1007/s00436-023-08087-9 38175278

[32] Stejskalova M Jancarova M Pruzinova K Capova K Varotto-Boccazzi I Epis S . Impact of *Asaia* bacteria on **Leishmania* major* development in sand flies: implications for vector control strategies. Parasit Vectors. (2025) 18:424. doi: 10.1186/s13071-025-07075-5 41126347 PMC12542282

[33] Ryan MP Pembroke JT . *Delftia* spp as Opportunistic Pathogens: a narrative review. New Microb New Infect. (2026) 70:101705. doi: 10.1016/j.nmni.2026.101705 41626520 PMC12857265

[34] Huang W Rodrigues J Bilgo E Tormo JR Challenger JD De Cozar-Gallardo C . *Delftia tsuruhatensis* TC1 symbiont suppresses malaria transmission by anopheline mosquitoes. Science. (2023) 381:533–40. doi: 10.1126/science.adf8141 37535741

[35] Vivero RJ Castañeda-Monsalve VA Romero LR G DH Cadavid-Restrepo G Moreno-Herrera CX . Gut microbiota dynamics in natural populations of *Pintomyia evansi* under experimental infection with **Leishmania* infantum*. Microorganisms. (2021) 9. doi: 10.3390/microorganisms9061214 34199688 PMC8228094

[36] Molina R . Laboratory adaptation of an autochthonous colony of *Phlebotomus perniciosus* Newstead 1911 (Diptera: Psychodidae). Res Rev Parasitol. (1991) 51:87–9.

[37] Molina R Jiménez M . Assessing the susceptibility to permethrin and deltamethrin of two laboratory strains of *Phlebotomus perniciosus* from Madrid region, Spain. Acta Trop. (2022) 231:106453. doi: 10.1016/j.actatropica.2022.106453 35430262

[38] Fraihi W Fares W Perrin P Dorkeld F Sereno D Barhoumi W . An integrated overview of the midgut bacterial flora composition of *Phlebotomus perniciosus*, a vector of zoonotic visceral leishmaniasis in the Western Mediterranean Basin. PloS NeglTrop Dis. (2017) 11:e0005484. doi: 10.1371/journal.pntd.0005484 28355207 PMC5386300

[39] Martín-Martín I Jiménez M González E Eguiluz C Molina R . Natural transmission of **Leishmania* infantum* through experimentally infected *Phlebotomus perniciosus* highlights the virulence of *Leishmania* parasites circulating in the human visceral leishmaniasis outbreak in Madrid, Spain. Vet Res. (2015) 46:138. doi: 10.1186/s13567-015-0281-1 PMC4673772 26645907

[40] Rutledge L Ward R Gould D . Studies on the feeding response of mosquitoes to nutritive solutions in a new membrane feeder. Mosq News. (1964) 24:407–9.

[41] Myskova J Votypka J Volf P . *Leishmania* in sand flies: comparison of quantitative polymerase chain reaction with other techniques to determine the intensity of infection. J Med Entomol. (2008) 45:133–38. doi: 10.1093/jmedent/45.1.133 18283954

[42] Agresti A . Categorical Data Analysis. New York, NY: John Wiley & Sons (2002).

[43] eCDC . Surveillance, Prevention and Control of *Leishmania*ses in the European Union and its Neighbouring Countries. Stockholm, Sweden: European Centre for Disease Prevention and Control (2022).

[44] Vaselek S . Overview of microbial studies in sandflies and their progress toward development of paratransgenic approach for the control of *Leishmania* sp. FrontTrop Dis. (2024) 5. doi: 10.3389/fitd.2024.1369077

[45] Tang K Zhang Y Meneses C Rogerio LA Willen L Iniguez E . *Phlebotomus duboscqi* gut microbiota dynamics in the context of *Leishmania* infection. Front Immunol. (2025) 16:1717935. doi: 10.3389/fimmu.2025.1717935 41562094 PMC12812887

[46] Qualls WA Müller GC Khallaayoune K Revay EE Zhioua E Kravchenko VD . Control of sand flies with attractive toxic sugar baits (ATSB) and potential impact on non-target organisms in Morocco. Parasit Vectors. (2015) 8:87. doi: 10.1186/s13071-015-0671-2 25890039 PMC4333173

[47] Omondi S Kosgei J Agumba S Polo B Yalla N Moshi V . Natural sugar feeding rates of *Anopheles* mosquitoes collected by different methods in western Kenya. Sci Rep. (2022) 12:20596. doi: 10.1038/s41598-022-25004-9 36446923 PMC9709062

[48] Moraes C Aguiar-Martins K Costa S Bates P Dillon R Genta F . Second blood meal by female *Lutzomyia longipalpis*: enhancement by oviposition and its effects on digestion, longevity, and *Leishmania* infection. BioMed Res Int. (2018) 2018:2472508. doi: 10.1155/2018/2472508 29770328 PMC5889884

[49] Schlein Y . Sandfly diet and *Leishmania*. Parasitol Today. (1986) 2:175–7. doi: 10.1016/0169-4758(86)90150-x 15462814

[50] Castro DP Genta FA Rogers ME . Exploiting nectar and blood feeding cues and phagostimulants to optimise Attractive Targeted Sugar Baits against a sand fly vector of leishmaniasis. PloS NeglTrop Dis. (2025) 19:e0013888. doi: 10.1371/journal.pntd.0013888 41452927 PMC12818734

[51] Hurwitz I Fieck A Read A Hillesland H Klein N Kang A . Paratransgenic control of vector borne diseases. Int J Biol Sci. (2011) 7:1334. doi: 10.7150/ijbs.7.1334 22110385 PMC3221369

