# Supplementary material for: Towards symbiont-based visceral leishmaniosis control: Delftia tsuruhatensis TC1 shows anti-Leishmania infantum effect in Phlebotomus perniciosus
Source: Front Immunol. 2026 Oct 30;17:1953324. doi: 10.3389/fimmu.2026.1953324 (PMC13759354; doi:10.3389/fimmu.2026.1953324)
Supplement: Supplementary file 1 [file DataSheet1.pdf]

#Evaluating Cochran–Armitage test for 3*2 contingency table from *Leishmania* infection data

```
library(DescTools)

Control_group <- c(0,1,21)

Study_group <- c(5,6,6)

mat <- matrix(c(Control_group, Study_group),

          nrow=2,

          byrow=TRUE)

colnames(TC1) <- c("No infection", "Immature infection", "Mature infection")

rownames(TC1) <- c("Control", "Study")

CochranArmitageTest(mat, alternative="two.sided")
```
