# Supplementary material for: Towards symbiont-based visceral leishmaniosis control: Delftia tsuruhatensis TC1 shows anti-Leishmania infantum effect in Phlebotomus perniciosus
Source: Front Immunol. 2026 Oct 30;17:1953324. doi: 10.3389/fimmu.2026.1953324 (PMC13759354; doi:10.3389/fimmu.2026.1953324)
Supplement: Supplementary file 2 [file DataSheet2.docx]

Supplementary Material

#Evaluating Cochran–Armitage test for 3*2 contingency table from *Leishmania* infection data

library(DescTools)

Control_group <- c(0,1,21)

Study_group <- c(5,6,6)

mat <- matrix(c(Control_group, Study_group),

nrow=2,

byrow=TRUE)

colnames(TC1) <- c("No infection", "Immature infection", "Mature infection")

rownames(TC1) <- c("Control", "Study")

CochranArmitageTest(mat, alternative="two.sided")

**Supplementary Data 1.** **R coding for evaluating the lineal tendency of *Leishmania*-infection maturity between control and TC1-fed sand flies.** Number of sand flies within each maturity level is presented as the vectors Control_group and Study_group (TC1-fed sand flies). For code rerunning with different data, modify vector values with obtained infection maturity data.

**Supplementary Figure 1.** **TC1 midgut-colonization through spiked-sugar meals at different TC1 doses of adult female *Phlebotomus perniciosus* (*Ppern*-BOA). A.** Experimental workflow. **B-C.** TC1 colonization rate. Dots and bar-plots indicate median values with ±95% IC of median colony forming units (CFU) per single dissected female (**B**) or median TC1 prevalence per assay (**C**) per days post TC1 ingestion (X-axis). Data was grouped per TC1 dose (blue squares, 10^8^ CFU/ml; purple circles, 10^10^ CFU/ml). Data from 2-3 independent replicates are shown. Non-parametric paired Mann-Whitney (**B**) and Friedman (**C**) tests are presented above bars (ns, non-significant; *, p<0.05; **, *p*<0.01; 0.06, obtained *p-value*). Descriptive values are shown below each graph: dpsm, days post sugar meal; n assays, number of experiments included in the analysis; n sand flies, total number of individuals included in each analysis; Median, median values; N/A, not available. Infographic created with BioRender.com.

**Supplementary Figure 2. TC1 midgut-colonization through spiked-sugar meals of adult female *Phlebotomus perniciosus* (*Ppern*-BOA) independent assays**. Dots represent TC1 abundance (CFU/midgut, Y-axis) per individually dissected and plated Ppern-BOA midguts at different days-post TC1- feeding at 10^8^ (**A-C**) and 10^10^ (**D-E**) CFU/ml (X-axis). Descriptive values are presented.

**Supplementary Figure 3. Probability of female sand fly survival (Y-axis) per day since spiked-sugar feeding (X-axis).** **A**. Survival rate sand flies fed a blood meal spiked with either 50% TC1-supernatant (SUP; blue straight-line) or 50% M9 medium (grey dotted-line). **B**. Survival rate of sand flies fed a blood meal spiked with either 100 µM harmane (blue straight-line) or 10% DMSO (grey dotted-line). **A-B**. Kaplan Meier test; ns, non-significant differences.

**Supplementary Figure 4. Additional TC1 anti-*Leishmania infantum* (BOS1FL1) *in vivo* effect in *Phlebotomus perniciosus* (*Ppern*-BOA and *Ppern*-FUE) assays.** Dots represent *L. infantum*-promastigote count per midgut between sand flies from the different study groups: non-bacteria colonized sugar-feeding (Control, gray), TC1-spiked (10^8^ CFU/ml, blue), and *Pantoea dispersa*-spiked (10^8^ CFU/ml, pink). The downward red arrow represents the percentage reduction of the *Leishmania*-promastigote count of TC1-colonized compared to *Pa. dispersa*-colonized females. Kruskal-Wallis t-test: ****, *p*<0.0001; ns, non-significant. Presented data include a preliminary assay conducted with *Ppern*-BOA colony (**A**) and assays conducted with *Ppern*-FUE either preliminary (**B**) or final (**C**).

**Supplementary Table 1. *In vitro* inhibition of *Leishmania infantum* by harmane.** Results are summarized by *L. infantum* strain (BOS1FL1, POL2FL6 and 28840MO), number of assays, and reading time. h, hours after plate setup. Original and log-transformed IC50 and R² values are shown.
